# Supplementary material for: Comparative genomics provides new insights into the diversity, physiology, and sexuality of the only industrially exploited tremellomycete: Phaffia rhodozyma
Source: BMC Genomics. 2016 Nov 9;17:901. doi: 10.1186/s12864-016-3244-7 (PMC5103461; doi:10.1186/s12864-016-3244-7)
Supplement: Additional file 6: — List of orphan genes with links to PFAM (related to Additional file 1: Table S1). (ZIP 1428 kb) [file 12864_2016_3244_MOESM6_ESM.zip › BLAST_HTML_FTR/G01183_P.html]

BLAST Search Results


```
BLASTP 2.2.27+


Reference:
Stephen F. Altschul, Thomas L. Madden, Alejandro A. Schäffer,
Jinghui Zhang, Zheng Zhang, Webb Miller, and David J. Lipman (1997),
"Gapped BLAST and PSI-BLAST: a new generation of protein database
search programs", Nucleic Acids Res. 25:3389-3402.


Reference for
composition-based statistics:
Alejandro A. Schäffer, L. Aravind, Thomas L. Madden, Sergei
Shavirin, John L. Spouge, Yuri I. Wolf, Eugene V. Koonin, and
Stephen F. Altschul (2001), "Improving the accuracy of PSI-BLAST
protein database searches with composition-based statistics and
other refinements", Nucleic Acids Res. 29:2994-3005.


Database: nr
           71,551,133 sequences; 26,053,659,533 total letters


Query= G01183_P

Length=381
                                                                      Score     E
Sequences producing significant alignments:                          (Bits)  Value

emb|CED82921.1|  hypothetical protein [Xanthophyllomyces dendrorh...   769    0.0  
ref|XP_006107076.1|  PREDICTED: HLA class I histocompatibility an...  42.4    0.55 
gb|ADE61506.1|  MHC class I antigen [Equus asinus]                    38.9    2.0  
dbj|BAA20881.1|  HLA-A26 [Homo sapiens]                               39.7    2.8  
gb|AAB97483.1|  MHC class I Caja-G*04, partial [Callithrix jacchus]   40.0    3.4  
gb|AGT56444.1|  MHC class I antigen, partial [Callithrix jacchus]     39.7    3.5  
ref|XP_007801100.1|  hypothetical protein EPUS_03160 [Endocarpon ...  40.0    3.5  
gb|AGW23420.1|  MHC class I antigen, partial [Callithrix jacchus]     39.7    3.5  
emb|CCH51024.2|  MHC class I antigen, partial [Callithrix jacchus]    40.0    3.6  
gb|AEG89318.1|  MHC class I antigen [Macaca nemestrina]               38.9    3.6  
gb|AGT56441.1|  MHC class I antigen, partial [Callithrix jacchus]     39.7    3.7  
emb|CCH51045.2|  MHC class I antigen, partial [Callithrix jacchus]    40.0    3.7  
gb|AGT56438.1|  MHC class I antigen, partial [Callithrix jacchus]     39.7    3.8  
gb|AEG89337.1|  MHC class I antigen [Macaca nemestrina]               39.3    4.4  
gb|AGT56427.1|  MHC class I antigen, partial [Callithrix jacchus]     38.9    6.0  
gb|AHI59038.1|  MHC class I antigen, partial [Macaca mulatta]         38.9    6.3  
gb|ADQ19530.1|  MHC class I antigen, partial [Macaca fascicularis]    38.1    6.4  
gb|ADE21911.1|  MHC class I antigen [Macaca mulatta]                  39.3    6.8  
gb|ADQ19537.1|  MHC class I antigen, partial [Macaca fascicularis]    38.1    6.8  
gb|ABU87869.1|  MHC class I antigen [Macaca mulatta] >dbj|BAH8538...  38.9    7.1  
emb|CRL08942.1|  MHC class I protein [Macaca fascicularis]            38.9    7.2  
gb|ADN95119.1|  MHC class I heavy chain antigen [Macaca fascicula...  38.5    7.5  
gb|ACS34837.1|  MHC class I antigen [Macaca mulatta]                  38.5    7.6  
emb|CAR82175.1|  MHC class I antigen [Macaca fascicularis]            38.9    7.8  
gb|AGS56967.1|  MHC class I antigen, partial [Ailuropoda melanole...  38.1    7.9  
gb|AJZ74052.1|  MHC class I antigen [Cercocebus atys]                 38.9    7.9  
gb|AHI59082.1|  MHC class I antigen, partial [Macaca mulatta]         38.5    8.0  
dbj|BAJ06159.1|  MHC class I antigen [Macaca mulatta]                 38.1    8.2  
gb|ACS34885.1|  MHC class I antigen [Macaca fascicularis] >gb|ADQ...  38.5    8.3  
gb|ACS34887.1|  MHC class I antigen [Macaca fascicularis]             38.5    8.4  
gb|AHI59081.1|  MHC class I antigen, partial [Macaca mulatta]         38.5    8.5  
emb|CDP90172.1|  MHC class I antigen, partial [Macaca fascicularis]   38.5    8.6  
emb|CAP16798.1|  MHC class I antigen, partial [Macaca mulatta]        38.9    8.7  
gb|ADQ19522.1|  MHC class I antigen, partial [Macaca fascicularis...  37.7    8.7  
gb|ADN95047.1|  MHC class I heavy chain antigen [Macaca fascicula...  38.5    8.8  
gb|ACS34886.1|  MHC class I antigen [Macaca fascicularis]             38.5    9.5  
emb|CRL08910.1|  MHC class I protein [Macaca fascicularis]            38.5    9.6  
gb|ACV04046.2|  MHC class I antigen [Macaca nemestrina]               37.7    9.6  
gb|ADQ19549.1|  MHC class I antigen, partial [Macaca fascicularis]    37.7    9.6  
dbj|BAJ06166.1|  MHC class I antigen [Macaca mulatta]                 38.1    9.8  
gb|ADO51729.1|  MHC class I antigen heavy chain [Macaca fascicula...  38.5    9.9  
gb|AEG89315.1|  MHC class I antigen [Macaca nemestrina]               38.1    9.9  
dbj|BAK74794.1|  MHC class I antigen, partial [Macaca fascicularis]   38.1    9.9  


 >emb|CED82921.1| hypothetical protein [Xanthophyllomyces dendrorhous]
Length=380

 Score =  769 bits (1987),  Expect = 0.0, Method: Compositional matrix adjust.
 Identities = 380/380 (100%), Positives = 380/380 (100%), Gaps = 0/380 (0%)

Query  1    MPFQTKRTYSESSLSPPSSAEEDDGIRSPSPPRKRPFVSGSDPSTAQSASDGFSSLFMNG  60
            MPFQTKRTYSESSLSPPSSAEEDDGIRSPSPPRKRPFVSGSDPSTAQSASDGFSSLFMNG
Sbjct  1    MPFQTKRTYSESSLSPPSSAEEDDGIRSPSPPRKRPFVSGSDPSTAQSASDGFSSLFMNG  60

Query  61   ARKRPACDSDLEGDDCDLGISSDEQEITGQNPWSSDSPSGFSSGNLNSHPWKTSPFLGSR  120
            ARKRPACDSDLEGDDCDLGISSDEQEITGQNPWSSDSPSGFSSGNLNSHPWKTSPFLGSR
Sbjct  61   ARKRPACDSDLEGDDCDLGISSDEQEITGQNPWSSDSPSGFSSGNLNSHPWKTSPFLGSR  120

Query  121  PEDGSSRQREQPLRKRTKRGLSEGFNQLGISEDLRTPLIEEEQPGFIHPSQQSAQQFAFP  180
            PEDGSSRQREQPLRKRTKRGLSEGFNQLGISEDLRTPLIEEEQPGFIHPSQQSAQQFAFP
Sbjct  121  PEDGSSRQREQPLRKRTKRGLSEGFNQLGISEDLRTPLIEEEQPGFIHPSQQSAQQFAFP  180

Query  181  RAGNLGNNIGEVNNNEGDLQPSSVVEPGEVGPAVWLGHAQNDDASDGLRFTVLNEDGNEE  240
            RAGNLGNNIGEVNNNEGDLQPSSVVEPGEVGPAVWLGHAQNDDASDGLRFTVLNEDGNEE
Sbjct  181  RAGNLGNNIGEVNNNEGDLQPSSVVEPGEVGPAVWLGHAQNDDASDGLRFTVLNEDGNEE  240

Query  241  PKLSRQSNQEDWKVGVWKGKNRDQGPGRLYVHPSYMNQHPSHSSYLPSSHPWDPNQPHKD  300
            PKLSRQSNQEDWKVGVWKGKNRDQGPGRLYVHPSYMNQHPSHSSYLPSSHPWDPNQPHKD
Sbjct  241  PKLSRQSNQEDWKVGVWKGKNRDQGPGRLYVHPSYMNQHPSHSSYLPSSHPWDPNQPHKD  300

Query  301  MILYNPLNNSQKLLEPSSPTSSRSSFLSSNTPIDAMMDTELYSNPNGPSYQQDWFGNGPR  360
            MILYNPLNNSQKLLEPSSPTSSRSSFLSSNTPIDAMMDTELYSNPNGPSYQQDWFGNGPR
Sbjct  301  MILYNPLNNSQKLLEPSSPTSSRSSFLSSNTPIDAMMDTELYSNPNGPSYQQDWFGNGPR  360

Query  361  FEEIADDEPSPEAGGGMDID  380
            FEEIADDEPSPEAGGGMDID
Sbjct  361  FEEIADDEPSPEAGGGMDID  380


>ref|XP_006107076.1| PREDICTED: HLA class I histocompatibility antigen, A-36 alpha 
chain-like, partial [Myotis lucifugus]
Length=306

 Score = 42.4 bits (98),  Expect = 0.55, Method: Compositional matrix adjust.
 Identities = 30/111 (27%), Positives = 47/111 (42%), Gaps = 2/111 (2%)

Query  154  LRTPLIEEEQPGFIHPSQQSAQQFAFPRAGNLGNNIGEVNNNEGDLQPSSVVEPGEVGPA  213
            ++ P +E+E PG+       A+  A    GNL N  G  N +E       V+   ++GP 
Sbjct  76   MQQPWVEQEDPGYWEEQTLKAKDTAQLFRGNLQNLRGYYNQSEDGSHTLQVMFGCDIGPE  135

Query  214  VWLGHAQNDDASDGLRFTVLNEDGN--EEPKLSRQSNQEDWKVGVWKGKNR  262
              L      DA DG+ +  LNED        ++ Q  +  W+   W  + R
Sbjct  136  GSLLRGYRQDAYDGVDYIALNEDLTSWTAADMAAQITKHKWEAEGWAERWR  186


>gb|ADE61506.1| MHC class I antigen [Equus asinus]
Length=137

 Score = 38.9 bits (89),  Expect = 2.0, Method: Compositional matrix adjust.
 Identities = 27/82 (33%), Positives = 35/82 (43%), Gaps = 0/82 (0%)

Query  155  RTPLIEEEQPGFIHPSQQSAQQFAFPRAGNLGNNIGEVNNNEGDLQPSSVVEPGEVGPAV  214
            R P +E+E P +     Q A   A    GNL N  G  N +E       ++   +VGP  
Sbjct  30   RAPWMEQEGPEYWEEETQRAAGLAHSFRGNLNNLRGYYNQSEAGSHTLQLMYGCDVGPHG  89

Query  215  WLGHAQNDDASDGLRFTVLNED  236
             L  A    A DG  +  LNED
Sbjct  90   RLLSASFQYAYDGADYLALNED  111


>dbj|BAA20881.1| HLA-A26 [Homo sapiens]
Length=213

 Score = 39.7 bits (91),  Expect = 2.8, Method: Compositional matrix adjust.
 Identities = 33/130 (25%), Positives = 47/130 (36%), Gaps = 16/130 (12%)

Query  155  RTPLIEEEQPGFIHPSQQSAQQFAFPRAGNLGNNIGEVNNNEGDLQPSSVVEPGEVGPAV  214
            R P IE+E P +   + ++ +  +     NLG   G  N +E        +   +VGP  
Sbjct  72   RAPWIEQEGPEYWDRNTRNVKAHSQTDRANLGTLRGYYNQSEDGSHTIQRMYGCDVGPDG  131

Query  215  WLGHAQNDDASDGLRFTVLNEDGN----------------EEPKLSRQSNQEDWKVGVWK  258
                    DA DG  +  LNED                  E P + R S +  W+ G W 
Sbjct  132  RFLRGYQQDAYDGKDYIALNEDLRSWTAADMAAQITQRKWETPPMRRSSGEPTWRAGAWS  191

Query  259  GKNRDQGPGR  268
            G       GR
Sbjct  192  GSADTWRTGR  201


>gb|AAB97483.1| MHC class I Caja-G*04, partial [Callithrix jacchus]
Length=356

 Score = 40.0 bits (92),  Expect = 3.4, Method: Compositional matrix adjust.
 Identities = 28/103 (27%), Positives = 41/103 (40%), Gaps = 2/103 (2%)

Query  155  RTPLIEEEQPGFIHPSQQSAQQFAFPRAGNLGNNIGEVNNNEGDLQPSSVVEPGEVGPAV  214
            R P +E+E P +     + A+  A    GNL   +G  N  +        +   +VGP  
Sbjct  64   RAPWVEQEGPEYWDEETRRAKTLAQTFRGNLRTALGYYNQTDAGSHTIQWMSGCDVGPDG  123

Query  215  WLGHAQNDDASDGLRFTVLNEDGN--EEPKLSRQSNQEDWKVG  255
             L    N  A DG  +  LNED      P ++ Q  Q  W+  
Sbjct  124  RLLRGYNQFAYDGKDYIALNEDLRSWTAPDVAAQITQRKWEAA  166


>gb|AGT56444.1| MHC class I antigen, partial [Callithrix jacchus]
Length=290

 Score = 39.7 bits (91),  Expect = 3.5, Method: Compositional matrix adjust.
 Identities = 28/103 (27%), Positives = 41/103 (40%), Gaps = 2/103 (2%)

Query  155  RTPLIEEEQPGFIHPSQQSAQQFAFPRAGNLGNNIGEVNNNEGDLQPSSVVEPGEVGPAV  214
            R P +E+E P +     + A+  A    GNL   +G  N  +        +   +VGP  
Sbjct  64   RAPWVEQEGPEYWDEETRRAKTLAQTFRGNLRTALGYYNQTDAGSHTIQWMSGCDVGPDG  123

Query  215  WLGHAQNDDASDGLRFTVLNEDGN--EEPKLSRQSNQEDWKVG  255
             L    N  A DG  +  LNED      P ++ Q  Q  W+  
Sbjct  124  RLLRGYNQFAYDGKDYIALNEDLRSWTAPDVAAQITQRKWEAA  166


>ref|XP_007801100.1| hypothetical protein EPUS_03160 [Endocarpon pusillum Z07020]
 gb|ERF73327.1| hypothetical protein EPUS_03160 [Endocarpon pusillum Z07020]
Length=396

 Score = 40.0 bits (92),  Expect = 3.5, Method: Compositional matrix adjust.
 Identities = 32/93 (34%), Positives = 41/93 (44%), Gaps = 9/93 (10%)

Query  52   GFSSLFMNGARKRPACDSDLEGDDCDLGISSDEQEITGQNPWSSDSPSGFSSGNLNSHPW  111
             +S +  N   +R A    LE   C  GI        G++ W SD   GF  G +   P 
Sbjct  211  AYSGVVANSVTRRYA----LENGLCKEGIVLKSSLKNGEDCWLSDGVRGFFQGTICLDP-  265

Query  112  KTSPFLGSRPEDGSSRQREQPLRKRTKRGLSEG  144
              S   GSRPE  S+R R  P  KR ++  SEG
Sbjct  266  --STLHGSRPEAHSNRDRAIP--KRDEKIRSEG  294


>gb|AGW23420.1| MHC class I antigen, partial [Callithrix jacchus]
Length=290

 Score = 39.7 bits (91),  Expect = 3.5, Method: Compositional matrix adjust.
 Identities = 28/103 (27%), Positives = 41/103 (40%), Gaps = 2/103 (2%)

Query  155  RTPLIEEEQPGFIHPSQQSAQQFAFPRAGNLGNNIGEVNNNEGDLQPSSVVEPGEVGPAV  214
            R P +E+E P +     + A+  A    GNL   +G  N  +        +   +VGP  
Sbjct  64   RAPWVEQEGPEYWDEETRRAKTLAQTFRGNLRTALGYYNQTDAGSHTIQWMSGCDVGPDG  123

Query  215  WLGHAQNDDASDGLRFTVLNEDGN--EEPKLSRQSNQEDWKVG  255
             L    N  A DG  +  LNED      P ++ Q  Q  W+  
Sbjct  124  RLLRGYNQFAYDGKDYIALNEDLRSWTAPDVAAQITQRKWEAA  166


>emb|CCH51024.2| MHC class I antigen, partial [Callithrix jacchus]
Length=363

 Score = 40.0 bits (92),  Expect = 3.6, Method: Compositional matrix adjust.
 Identities = 28/103 (27%), Positives = 41/103 (40%), Gaps = 2/103 (2%)

Query  155  RTPLIEEEQPGFIHPSQQSAQQFAFPRAGNLGNNIGEVNNNEGDLQPSSVVEPGEVGPAV  214
            R P +E+E P +     + A+  A    GNL   +G  N  +        +   +VGP  
Sbjct  71   RAPWVEQEGPEYWDEETRRAKTLAQTFRGNLRTALGYYNQTDAGSHTIQWMSGCDVGPDG  130

Query  215  WLGHAQNDDASDGLRFTVLNEDGN--EEPKLSRQSNQEDWKVG  255
             L    N  A DG  +  LNED      P ++ Q  Q  W+  
Sbjct  131  RLLRGYNQFAYDGKDYIALNEDLRSWTAPDVAAQITQRKWEAA  173


>gb|AEG89318.1| MHC class I antigen [Macaca nemestrina]
Length=177

 Score = 38.9 bits (89),  Expect = 3.6, Method: Compositional matrix adjust.
 Identities = 27/103 (26%), Positives = 45/103 (44%), Gaps = 2/103 (2%)

Query  155  RTPLIEEEQPGFIHPSQQSAQQFAFPRAGNLGNNIGEVNNNEGDLQPSSVVEPGEVGPAV  214
            R P +E+E P +   + +SA+  A    GNL   +   N +EG      ++   ++GP  
Sbjct  18   RAPWMEQEGPEYWDRNTRSAKGHAQTHRGNLRTLLRYYNQSEGGSHTVQIMSGCDLGPDG  77

Query  215  WLGHAQNDDASDGLRFTVLNEDGN--EEPKLSRQSNQEDWKVG  255
             L    +  A DG  +  LNED        ++ Q+ Q  W+  
Sbjct  78   RLLRGYHQHAYDGKDYIALNEDLRSWTAADMAAQNTQRKWEAA  120


>gb|AGT56441.1| MHC class I antigen, partial [Callithrix jacchus]
Length=290

 Score = 39.7 bits (91),  Expect = 3.7, Method: Compositional matrix adjust.
 Identities = 28/103 (27%), Positives = 41/103 (40%), Gaps = 2/103 (2%)

Query  155  RTPLIEEEQPGFIHPSQQSAQQFAFPRAGNLGNNIGEVNNNEGDLQPSSVVEPGEVGPAV  214
            R P +E+E P +     + A+  A    GNL   +G  N  +        +   +VGP  
Sbjct  64   RAPWVEQEGPEYWDEETRRAKTLAQTFRGNLRTALGYYNQTDAGSHTIQWMSGCDVGPDG  123

Query  215  WLGHAQNDDASDGLRFTVLNEDGN--EEPKLSRQSNQEDWKVG  255
             L    N  A DG  +  LNED      P ++ Q  Q  W+  
Sbjct  124  RLLRGYNQFAYDGKDYIALNEDLRSWTAPDVAAQITQRKWEAA  166


>emb|CCH51045.2| MHC class I antigen, partial [Callithrix jacchus]
Length=363

 Score = 40.0 bits (92),  Expect = 3.7, Method: Compositional matrix adjust.
 Identities = 28/103 (27%), Positives = 41/103 (40%), Gaps = 2/103 (2%)

Query  155  RTPLIEEEQPGFIHPSQQSAQQFAFPRAGNLGNNIGEVNNNEGDLQPSSVVEPGEVGPAV  214
            R P +E+E P +     + A+  A    GNL   +G  N  +        +   +VGP  
Sbjct  71   RAPWVEQEGPEYWDEETRRAKTLAQTFRGNLRTALGYYNQTDAGSHTIQWMSGCDVGPDG  130

Query  215  WLGHAQNDDASDGLRFTVLNEDGN--EEPKLSRQSNQEDWKVG  255
             L    N  A DG  +  LNED      P ++ Q  Q  W+  
Sbjct  131  RLLRGYNQFAYDGKDYIALNEDLRSWTAPDVAAQITQRKWEAA  173


>gb|AGT56438.1| MHC class I antigen, partial [Callithrix jacchus]
Length=290

 Score = 39.7 bits (91),  Expect = 3.8, Method: Compositional matrix adjust.
 Identities = 28/103 (27%), Positives = 41/103 (40%), Gaps = 2/103 (2%)

Query  155  RTPLIEEEQPGFIHPSQQSAQQFAFPRAGNLGNNIGEVNNNEGDLQPSSVVEPGEVGPAV  214
            R P +E+E P +     + A+  A    GNL   +G  N  +        +   +VGP  
Sbjct  64   RAPWVEQEGPEYWDEETRRAKTLAQTFRGNLRTALGYYNQTDAGSHTIQWMSGCDVGPDG  123

Query  215  WLGHAQNDDASDGLRFTVLNEDGN--EEPKLSRQSNQEDWKVG  255
             L    N  A DG  +  LNED      P ++ Q  Q  W+  
Sbjct  124  RLLRGYNQFAYDGKDYIALNEDLRSWTAPDVAAQITQRKWEAA  166


>gb|AEG89337.1| MHC class I antigen [Macaca nemestrina]
Length=231

 Score = 39.3 bits (90),  Expect = 4.4, Method: Compositional matrix adjust.
 Identities = 29/103 (28%), Positives = 43/103 (42%), Gaps = 2/103 (2%)

Query  155  RTPLIEEEQPGFIHPSQQSAQQFAFPRAGNLGNNIGEVNNNEGDLQPSSVVEPGEVGPAV  214
            R+  IE+E P +     + A+  A     NLG   G  N +EG      ++   ++GP  
Sbjct  72   RSWWIEQEGPEYWEEETRKAKARAQTDRVNLGTLRGYYNQSEGGSHTIQIMYGCDLGPDG  131

Query  215  WLGHAQNDDASDGLRFTVLNEDGN--EEPKLSRQSNQEDWKVG  255
             L      DA DG  +  LNED        ++ QS Q  W+  
Sbjct  132  RLLRGYRQDAYDGKDYIALNEDLRSWTAADMAAQSTQRKWEAA  174


>gb|AGT56427.1| MHC class I antigen, partial [Callithrix jacchus]
Length=270

 Score = 38.9 bits (89),  Expect = 6.0, Method: Compositional matrix adjust.
 Identities = 28/103 (27%), Positives = 41/103 (40%), Gaps = 2/103 (2%)

Query  155  RTPLIEEEQPGFIHPSQQSAQQFAFPRAGNLGNNIGEVNNNEGDLQPSSVVEPGEVGPAV  214
            R P +E+E P +     + A+  A    GNL   +G  N  +        +   +VGP  
Sbjct  64   RAPWVEQEGPEYWDEETRRAKTLAQTFRGNLRTALGYYNQTDAGSHTIQWMSGCDVGPDG  123

Query  215  WLGHAQNDDASDGLRFTVLNEDGN--EEPKLSRQSNQEDWKVG  255
             L    N  A DG  +  LNED      P ++ Q  Q  W+  
Sbjct  124  RLLRGYNQFAYDGKDYIALNEDLRSWTAPDVAAQIAQRKWEAA  166


>gb|AHI59038.1| MHC class I antigen, partial [Macaca mulatta]
Length=231

 Score = 38.9 bits (89),  Expect = 6.3, Method: Compositional matrix adjust.
 Identities = 28/101 (28%), Positives = 44/101 (44%), Gaps = 2/101 (2%)

Query  155  RTPLIEEEQPGFIHPSQQSAQQFAFPRAGNLGNNIGEVNNNEGDLQPSSVVEPGEVGPAV  214
            R P +E+E P +     + A+  A     +LG   G  N +EG       +   ++GP  
Sbjct  72   RAPWVEQEGPEYWEEQTRIAKANAQTDRVDLGTLRGYYNQSEGGSHTIQWMYGCDLGPDG  131

Query  215  WLGHAQNDDASDGLRFTVLNEDGN--EEPKLSRQSNQEDWK  253
             L H  + DA DG  +  LNED        ++ Q+ Q  W+
Sbjct  132  RLLHGYHQDAYDGKDYIALNEDLRSWTAADIAAQNTQRKWE  172


>gb|ADQ19530.1| MHC class I antigen, partial [Macaca fascicularis]
Length=181

 Score = 38.1 bits (87),  Expect = 6.4, Method: Compositional matrix adjust.
 Identities = 29/104 (28%), Positives = 46/104 (44%), Gaps = 2/104 (2%)

Query  152  EDLRTPLIEEEQPGFIHPSQQSAQQFAFPRAGNLGNNIGEVNNNEGDLQPSSVVEPGEVG  211
            E+ R P +E+E P +     + A+  A     +LG   G  N +EG       +   ++G
Sbjct  44   EEPRAPWVEQEGPEYWEEQTRIAKANAQTDRVDLGTLRGYYNQSEGGSHTIQWMYGCDLG  103

Query  212  PAVWLGHAQNDDASDGLRFTVLNEDGN--EEPKLSRQSNQEDWK  253
            P   L H  + DA DG  +  LNED        ++ Q+ Q  W+
Sbjct  104  PDGRLLHGYHQDAYDGKDYIALNEDLRSWTAADIAAQNTQRKWE  147


>gb|ADE21911.1| MHC class I antigen [Macaca mulatta]
Length=359

 Score = 39.3 bits (90),  Expect = 6.8, Method: Compositional matrix adjust.
 Identities = 29/106 (27%), Positives = 44/106 (42%), Gaps = 2/106 (2%)

Query  152  EDLRTPLIEEEQPGFIHPSQQSAQQFAFPRAGNLGNNIGEVNNNEGDLQPSSVVEPGEVG  211
            E+ R P +E+E P +     + A+  A     NLG  +   N +EG       +   ++G
Sbjct  66   EEPRAPWVEQEGPEYWEEQTRIAKDTAQTDRANLGTLLRYYNQSEGGSHTLQWMYGCDLG  125

Query  212  PAVWLGHAQNDDASDGLRFTVLNEDGN--EEPKLSRQSNQEDWKVG  255
            P   L      DA DG  +  LNED        ++ Q+ Q  WK  
Sbjct  126  PDGRLLRGYRQDAYDGKDYIALNEDLRSWTAADVAAQNTQRKWKAA  171


>gb|ADQ19537.1| MHC class I antigen, partial [Macaca fascicularis]
Length=181

 Score = 38.1 bits (87),  Expect = 6.8, Method: Compositional matrix adjust.
 Identities = 29/103 (28%), Positives = 43/103 (42%), Gaps = 2/103 (2%)

Query  155  RTPLIEEEQPGFIHPSQQSAQQFAFPRAGNLGNNIGEVNNNEGDLQPSSVVEPGEVGPAV  214
            R   IE+E P +   + + A+  A    G LGN  G  N +EG       +   +VGP  
Sbjct  47   RARWIEQEGPEYWEEATRRAKGHAQTDLGGLGNLRGYYNQSEGGSHTYQRMSGCDVGPDG  106

Query  215  WLGHAQNDDASDGLRFTVLNEDGN--EEPKLSRQSNQEDWKVG  255
             L    +  A DG  +  LNED        ++ Q+ Q  W+  
Sbjct  107  RLLRGYHQRAYDGKDYIALNEDLRSWTAADMAAQNTQRKWEAA  149


>gb|ABU87869.1| MHC class I antigen [Macaca mulatta]
 dbj|BAH85387.1| MHC class I antigen [Macaca mulatta]
Length=359

 Score = 38.9 bits (89),  Expect = 7.1, Method: Compositional matrix adjust.
 Identities = 29/106 (27%), Positives = 44/106 (42%), Gaps = 2/106 (2%)

Query  152  EDLRTPLIEEEQPGFIHPSQQSAQQFAFPRAGNLGNNIGEVNNNEGDLQPSSVVEPGEVG  211
            E+ R P +E+E P +     + A+  A     NLG  +   N +EG       +   ++G
Sbjct  66   EEPRAPWVEQEGPEYWEEQTRIAKDTAQTDRANLGTLLRYYNQSEGGSHTLQWMYGCDLG  125

Query  212  PAVWLGHAQNDDASDGLRFTVLNEDGN--EEPKLSRQSNQEDWKVG  255
            P   L      DA DG  +  LNED        ++ Q+ Q  WK  
Sbjct  126  PDGRLLRGYRQDAYDGKDYIALNEDLRSWTAADVAAQNTQRKWKAA  171


>emb|CRL08942.1| MHC class I protein [Macaca fascicularis]
Length=362

 Score = 38.9 bits (89),  Expect = 7.2, Method: Compositional matrix adjust.
 Identities = 29/106 (27%), Positives = 44/106 (42%), Gaps = 2/106 (2%)

Query  152  EDLRTPLIEEEQPGFIHPSQQSAQQFAFPRAGNLGNNIGEVNNNEGDLQPSSVVEPGEVG  211
            E+ R P +E+E P +     + A+  A     NLG  +   N +EG       +   ++G
Sbjct  69   EEPRAPWVEQEGPEYWEEQTRIAKDTAQTDRANLGTLLRYYNQSEGGSHTLQWMYGCDLG  128

Query  212  PAVWLGHAQNDDASDGLRFTVLNEDGN--EEPKLSRQSNQEDWKVG  255
            P   L      DA DG  +  LNED        ++ Q+ Q  WK  
Sbjct  129  PDGRLLRGYRQDAYDGKDYIALNEDLRSWTAADVAAQNTQRKWKAA  174


>gb|ADN95119.1| MHC class I heavy chain antigen [Macaca fascicularis]
Length=273

 Score = 38.5 bits (88),  Expect = 7.5, Method: Compositional matrix adjust.
 Identities = 29/104 (28%), Positives = 46/104 (44%), Gaps = 2/104 (2%)

Query  152  EDLRTPLIEEEQPGFIHPSQQSAQQFAFPRAGNLGNNIGEVNNNEGDLQPSSVVEPGEVG  211
            E+ R P +E+E P +     + A+  A     +LG   G  N +EG       +   ++G
Sbjct  44   EEPRAPWVEQEGPEYWEEQTRIAKANAQTDRVDLGTLRGYYNQSEGGSHTIQWMYGCDLG  103

Query  212  PAVWLGHAQNDDASDGLRFTVLNEDGN--EEPKLSRQSNQEDWK  253
            P   L H  + DA DG  +  LNED        ++ Q+ Q  W+
Sbjct  104  PDGRLLHGYHQDAYDGKDYIALNEDLRSWTAADIAAQNTQRKWE  147


>gb|ACS34837.1| MHC class I antigen [Macaca mulatta]
Length=273

 Score = 38.5 bits (88),  Expect = 7.6, Method: Compositional matrix adjust.
 Identities = 28/101 (28%), Positives = 44/101 (44%), Gaps = 2/101 (2%)

Query  155  RTPLIEEEQPGFIHPSQQSAQQFAFPRAGNLGNNIGEVNNNEGDLQPSSVVEPGEVGPAV  214
            R P +E+E P +     + A+  A     +LG   G  N +EG       +   ++GP  
Sbjct  47   RAPWVEQEGPEYWEEETRRAKANAQTDRVDLGTLRGYYNQSEGGSHTIQWMYGCDLGPDG  106

Query  215  WLGHAQNDDASDGLRFTVLNEDGN--EEPKLSRQSNQEDWK  253
             L H  + DA DG  +  LNED        ++ Q+ Q  W+
Sbjct  107  RLLHGYHQDAYDGKDYIALNEDLRSWTAADIAAQNTQRKWE  147


>emb|CAR82175.1| MHC class I antigen [Macaca fascicularis]
Length=362

 Score = 38.9 bits (89),  Expect = 7.8, Method: Compositional matrix adjust.
 Identities = 29/106 (27%), Positives = 44/106 (42%), Gaps = 2/106 (2%)

Query  152  EDLRTPLIEEEQPGFIHPSQQSAQQFAFPRAGNLGNNIGEVNNNEGDLQPSSVVEPGEVG  211
            E+ R P +E+E P +     + A+  A     NLG  +   N +EG       +   ++G
Sbjct  69   EEPRAPWVEQEGPEYWEEQTRIAKDTAQTDRANLGTLLRYYNQSEGGSHTLQWMYGCDLG  128

Query  212  PAVWLGHAQNDDASDGLRFTVLNEDGN--EEPKLSRQSNQEDWKVG  255
            P   L      DA DG  +  LNED        ++ Q+ Q  WK  
Sbjct  129  PDGRLLRGYRQDAYDGKDYIALNEDLRSWTAADVAAQNTQRKWKAA  174


>gb|AGS56967.1| MHC class I antigen, partial [Ailuropoda melanoleuca]
Length=181

 Score = 38.1 bits (87),  Expect = 7.9, Method: Compositional matrix adjust.
 Identities = 30/115 (26%), Positives = 45/115 (39%), Gaps = 2/115 (2%)

Query  155  RTPLIEEEQPGFIHPSQQSAQQFAFPRAGNLGNNIGEVNNNEGDLQPSSVVEPGEVGPAV  214
            R P IE+E P +     + A+  A    GNL   +   N +E        +   +VGP  
Sbjct  47   RAPWIEQEGPEYWDRETRGAKDTAQTFRGNLQTALRYYNQSEAGSHTIQRMYGCDVGPDG  106

Query  215  WLGHAQNDDASDGLRFTVLNEDGN--EEPKLSRQSNQEDWKVGVWKGKNRDQGPG  267
             L    + DA DG  +  LNED         + Q  +  W+   W  + R+   G
Sbjct  107  RLLRGYSQDAYDGADYIALNEDLRSWTAADTAAQITRRKWEAAGWAERYRNYVEG  161


>gb|AJZ74052.1| MHC class I antigen [Cercocebus atys]
Length=362

 Score = 38.9 bits (89),  Expect = 7.9, Method: Compositional matrix adjust.
 Identities = 25/82 (30%), Positives = 38/82 (46%), Gaps = 0/82 (0%)

Query  155  RTPLIEEEQPGFIHPSQQSAQQFAFPRAGNLGNNIGEVNNNEGDLQPSSVVEPGEVGPAV  214
            R P +E+E P +   + +SA+  A    GNL   +   N +EG      +V   ++GP  
Sbjct  72   RAPWMEQEGPEYWDRNTRSAKGHAQTHRGNLRTLLRYYNQSEGGSHTVQIVSGCDLGPDG  131

Query  215  WLGHAQNDDASDGLRFTVLNED  236
             L    +  A DG  +  LNED
Sbjct  132  RLLRGYHQHAYDGKDYIALNED  153


>gb|AHI59082.1| MHC class I antigen, partial [Macaca mulatta]
Length=228

 Score = 38.5 bits (88),  Expect = 8.0, Method: Compositional matrix adjust.
 Identities = 28/100 (28%), Positives = 43/100 (43%), Gaps = 2/100 (2%)

Query  155  RTPLIEEEQPGFIHPSQQSAQQFAFPRAGNLGNNIGEVNNNEGDLQPSSVVEPGEVGPAV  214
            R P +E+E P +     + A++ A    GNL    G  N +EG       +   ++GP  
Sbjct  69   RAPWMEQEGPEYWEEQTRIAKEAAQTERGNLRTLRGYYNQSEGGSHTLQWMYGCDLGPDG  128

Query  215  WLGHAQNDDASDGLRFTVLNEDGN--EEPKLSRQSNQEDW  252
             L    +  A DG  + VLNED        ++ Q+ Q  W
Sbjct  129  RLLRGYDQSAYDGRDYIVLNEDLRSWTAADMAAQNTQRKW  168


>dbj|BAJ06159.1| MHC class I antigen [Macaca mulatta]
Length=215

 Score = 38.1 bits (87),  Expect = 8.2, Method: Compositional matrix adjust.
 Identities = 28/100 (28%), Positives = 43/100 (43%), Gaps = 2/100 (2%)

Query  155  RTPLIEEEQPGFIHPSQQSAQQFAFPRAGNLGNNIGEVNNNEGDLQPSSVVEPGEVGPAV  214
            R P +E+E P +     + A++ A    GNL    G  N +EG       +   ++GP  
Sbjct  60   RAPWMEQEGPEYWEEQTRIAKEAAQTERGNLRTLRGYYNQSEGGSHTLQWMYGCDLGPDG  119

Query  215  WLGHAQNDDASDGLRFTVLNEDGN--EEPKLSRQSNQEDW  252
             L    +  A DG  + VLNED        ++ Q+ Q  W
Sbjct  120  RLLRGYDQSAYDGRDYIVLNEDLRSWTAADMAAQNTQRKW  159


>gb|ACS34885.1| MHC class I antigen [Macaca fascicularis]
 gb|ADQ19492.1| MHC class I antigen [Macaca fascicularis]
Length=273

 Score = 38.5 bits (88),  Expect = 8.3, Method: Compositional matrix adjust.
 Identities = 29/104 (28%), Positives = 46/104 (44%), Gaps = 2/104 (2%)

Query  152  EDLRTPLIEEEQPGFIHPSQQSAQQFAFPRAGNLGNNIGEVNNNEGDLQPSSVVEPGEVG  211
            E+ R P +E+E P +     + A+  A     +LG   G  N +EG       +   ++G
Sbjct  44   EEPRAPWVEQEGPEYWEEQTRIAKANAQTDRVDLGTLRGYYNQSEGGSHTIQWMYGCDLG  103

Query  212  PAVWLGHAQNDDASDGLRFTVLNEDGN--EEPKLSRQSNQEDWK  253
            P   L H  + DA DG  +  LNED        ++ Q+ Q  W+
Sbjct  104  PDGRLLHGYHQDAYDGKDYIALNEDLRSWTAADIAAQNTQRKWE  147


>gb|ACS34887.1| MHC class I antigen [Macaca fascicularis]
Length=273

 Score = 38.5 bits (88),  Expect = 8.4, Method: Compositional matrix adjust.
 Identities = 29/104 (28%), Positives = 46/104 (44%), Gaps = 2/104 (2%)

Query  152  EDLRTPLIEEEQPGFIHPSQQSAQQFAFPRAGNLGNNIGEVNNNEGDLQPSSVVEPGEVG  211
            E+ R P +E+E P +     + A+  A     +LG   G  N +EG       +   ++G
Sbjct  44   EEPRAPWVEQEGPEYWEEQTRIAKANAQTDRVDLGTLRGYYNQSEGGSHTIQWMYGCDLG  103

Query  212  PAVWLGHAQNDDASDGLRFTVLNEDGN--EEPKLSRQSNQEDWK  253
            P   L H  + DA DG  +  LNED        ++ Q+ Q  W+
Sbjct  104  PDGRLLHGYHQDAYDGKDYIALNEDLRSWTAADIAAQNTQRKWE  147


>gb|AHI59081.1| MHC class I antigen, partial [Macaca mulatta]
Length=228

 Score = 38.5 bits (88),  Expect = 8.5, Method: Compositional matrix adjust.
 Identities = 28/100 (28%), Positives = 43/100 (43%), Gaps = 2/100 (2%)

Query  155  RTPLIEEEQPGFIHPSQQSAQQFAFPRAGNLGNNIGEVNNNEGDLQPSSVVEPGEVGPAV  214
            R P +E+E P +     + A++ A    GNL    G  N +EG       +   ++GP  
Sbjct  69   RAPWMEQEGPEYWEEQTRIAKEAAQTERGNLRTLRGYYNQSEGGSHTLQWMYGCDLGPDG  128

Query  215  WLGHAQNDDASDGLRFTVLNEDGN--EEPKLSRQSNQEDW  252
             L    +  A DG  + VLNED        ++ Q+ Q  W
Sbjct  129  RLLRGYDQSAYDGRDYIVLNEDLRSWTAADMAAQNTQRKW  168


>emb|CDP90172.1| MHC class I antigen, partial [Macaca fascicularis]
Length=231

 Score = 38.5 bits (88),  Expect = 8.6, Method: Compositional matrix adjust.
 Identities = 24/82 (29%), Positives = 38/82 (46%), Gaps = 0/82 (0%)

Query  155  RTPLIEEEQPGFIHPSQQSAQQFAFPRAGNLGNNIGEVNNNEGDLQPSSVVEPGEVGPAV  214
            R P +E+E P +   + +SA+  A    GNL   +   N +EG      ++   ++GP  
Sbjct  72   RAPWMEQEGPEYWDRNTRSAKGHAQTHRGNLRTLLRYYNQSEGGSHTVQIMSGCDLGPDG  131

Query  215  WLGHAQNDDASDGLRFTVLNED  236
             L    +  A DG  +  LNED
Sbjct  132  RLLRGYHQHAYDGKDYIALNED  153


>emb|CAP16798.1| MHC class I antigen, partial [Macaca mulatta]
Length=351

 Score = 38.9 bits (89),  Expect = 8.7, Method: Compositional matrix adjust.
 Identities = 29/106 (27%), Positives = 44/106 (42%), Gaps = 2/106 (2%)

Query  152  EDLRTPLIEEEQPGFIHPSQQSAQQFAFPRAGNLGNNIGEVNNNEGDLQPSSVVEPGEVG  211
            E+ R P +E+E P +     + A+  A     NLG  +   N +EG       +   ++G
Sbjct  58   EEPRAPWVEQEGPEYWEEQTRIAKDTAQTDRANLGTLLRYYNQSEGGSHTLQWMYGCDLG  117

Query  212  PAVWLGHAQNDDASDGLRFTVLNEDGN--EEPKLSRQSNQEDWKVG  255
            P   L      DA DG  +  LNED        ++ Q+ Q  WK  
Sbjct  118  PDGRLLRGYRQDAYDGKDYIALNEDLRSWTAADVAAQNTQRKWKAA  163


>gb|ADQ19522.1| MHC class I antigen, partial [Macaca fascicularis]
 gb|ADQ19538.1| MHC class I antigen, partial [Macaca fascicularis]
Length=181

 Score = 37.7 bits (86),  Expect = 8.7, Method: Compositional matrix adjust.
 Identities = 28/100 (28%), Positives = 43/100 (43%), Gaps = 2/100 (2%)

Query  155  RTPLIEEEQPGFIHPSQQSAQQFAFPRAGNLGNNIGEVNNNEGDLQPSSVVEPGEVGPAV  214
            R P +E+E P +     + A++ A    GNL    G  N +EG       +   ++GP  
Sbjct  47   RAPWMEQEGPEYWEEQTRIAKEAAQTERGNLRTLRGYYNQSEGGSHTLQWMYGCDLGPDG  106

Query  215  WLGHAQNDDASDGLRFTVLNEDGN--EEPKLSRQSNQEDW  252
             L    +  A DG  + VLNED        ++ Q+ Q  W
Sbjct  107  RLLRGYDQSAYDGRDYIVLNEDLRSWTAADMAAQNTQRKW  146


>gb|ADN95047.1| MHC class I heavy chain antigen [Macaca fascicularis]
Length=273

 Score = 38.5 bits (88),  Expect = 8.8, Method: Compositional matrix adjust.
 Identities = 27/103 (26%), Positives = 44/103 (43%), Gaps = 2/103 (2%)

Query  155  RTPLIEEEQPGFIHPSQQSAQQFAFPRAGNLGNNIGEVNNNEGDLQPSSVVEPGEVGPAV  214
            R P +E+E P +   + + A+  A    GNL   +   N +EG      ++   ++GP  
Sbjct  47   RAPWMEQEGPEYWDRNTRRAKGHAQTHRGNLRTLLRYYNQSEGGSHTVQIMSGCDLGPDG  106

Query  215  WLGHAQNDDASDGLRFTVLNEDGN--EEPKLSRQSNQEDWKVG  255
             L    N  A DG  +  LNED        ++ Q+ Q  W+  
Sbjct  107  RLLRGYNQHAYDGKDYIALNEDLRSWTAADMAAQNTQRKWEAA  149


>gb|ACS34886.1| MHC class I antigen [Macaca fascicularis]
Length=273

 Score = 38.5 bits (88),  Expect = 9.5, Method: Compositional matrix adjust.
 Identities = 28/103 (27%), Positives = 42/103 (41%), Gaps = 2/103 (2%)

Query  155  RTPLIEEEQPGFIHPSQQSAQQFAFPRAGNLGNNIGEVNNNEGDLQPSSVVEPGEVGPAV  214
            R P +E+E P +     + A+  A    GNL    G  N +EG       +   ++GP  
Sbjct  47   RAPWMEQEGPEYWEEETRIAKANAQTERGNLRTLRGYYNQSEGGSHTIQTMYGCDLGPDR  106

Query  215  WLGHAQNDDASDGLRFTVLNEDGN--EEPKLSRQSNQEDWKVG  255
             L      DA DG  +  LNED        ++ Q+ Q  W+  
Sbjct  107  HLLRGYRQDAYDGRDYIALNEDLRSWTAADMAAQNTQRKWEAA  149


>emb|CRL08910.1| MHC class I protein [Macaca fascicularis]
Length=362

 Score = 38.5 bits (88),  Expect = 9.6, Method: Compositional matrix adjust.
 Identities = 29/104 (28%), Positives = 46/104 (44%), Gaps = 2/104 (2%)

Query  152  EDLRTPLIEEEQPGFIHPSQQSAQQFAFPRAGNLGNNIGEVNNNEGDLQPSSVVEPGEVG  211
            E+ R P +E+E P +     + A+  A     +LG   G  N +EG       +   ++G
Sbjct  69   EEPRAPWVEQEGPEYWEEQTRIAKANAQTDRVDLGTLRGYYNQSEGGSHTIQWMYGCDLG  128

Query  212  PAVWLGHAQNDDASDGLRFTVLNEDGN--EEPKLSRQSNQEDWK  253
            P   L H  + DA DG  +  LNED        ++ Q+ Q  W+
Sbjct  129  PDGRLLHGYHQDAYDGKDYIALNEDLRSWTAADIAAQNTQRKWE  172


>gb|ACV04046.2| MHC class I antigen [Macaca nemestrina]
Length=177

 Score = 37.7 bits (86),  Expect = 9.6, Method: Compositional matrix adjust.
 Identities = 24/82 (29%), Positives = 37/82 (45%), Gaps = 0/82 (0%)

Query  155  RTPLIEEEQPGFIHPSQQSAQQFAFPRAGNLGNNIGEVNNNEGDLQPSSVVEPGEVGPAV  214
            R P +E+E P +   + + A+  A    GNL   +   N +EG      ++   ++GP  
Sbjct  18   RAPWMEQEGPEYWDRNTRIAKGHAQTHRGNLRTLLRYYNQSEGGSHTVQIMSGCDLGPDG  77

Query  215  WLGHAQNDDASDGLRFTVLNED  236
             L    N  A DG  +  LNED
Sbjct  78   RLLRGYNQHAYDGKDYIALNED  99


>gb|ADQ19549.1| MHC class I antigen, partial [Macaca fascicularis]
Length=181

 Score = 37.7 bits (86),  Expect = 9.6, Method: Compositional matrix adjust.
 Identities = 28/101 (28%), Positives = 44/101 (44%), Gaps = 2/101 (2%)

Query  155  RTPLIEEEQPGFIHPSQQSAQQFAFPRAGNLGNNIGEVNNNEGDLQPSSVVEPGEVGPAV  214
            R P +E+E P +     + A+  A     +LG   G  N +EG       +   ++GP  
Sbjct  47   RAPWMEQEGPEYWEEQTRIAKANAQTDRVDLGTLRGYYNQSEGGSHTIQWMYGCDLGPDG  106

Query  215  WLGHAQNDDASDGLRFTVLNEDGN--EEPKLSRQSNQEDWK  253
             L H  + DA DG  +  LNED        ++ Q+ Q  W+
Sbjct  107  RLLHGYHQDAYDGKDYIALNEDLRSWTAADIAAQNTQRKWE  147


>dbj|BAJ06166.1| MHC class I antigen [Macaca mulatta]
Length=214

 Score = 38.1 bits (87),  Expect = 9.8, Method: Compositional matrix adjust.
 Identities = 24/82 (29%), Positives = 38/82 (46%), Gaps = 0/82 (0%)

Query  155  RTPLIEEEQPGFIHPSQQSAQQFAFPRAGNLGNNIGEVNNNEGDLQPSSVVEPGEVGPAV  214
            R P +E+E P +   + +SA+  A    GNL   +   N +EG      ++   ++GP  
Sbjct  60   RAPWMEQEGPEYWDRNTRSAKGHAQTHRGNLRTLLRYYNQSEGGSHTVQIMSGCDLGPDG  119

Query  215  WLGHAQNDDASDGLRFTVLNED  236
             L    +  A DG  +  LNED
Sbjct  120  RLLRGYHQHAYDGKDYIALNED  141


>gb|ADO51729.1| MHC class I antigen heavy chain [Macaca fascicularis]
Length=273

 Score = 38.5 bits (88),  Expect = 9.9, Method: Compositional matrix adjust.
 Identities = 29/104 (28%), Positives = 46/104 (44%), Gaps = 2/104 (2%)

Query  152  EDLRTPLIEEEQPGFIHPSQQSAQQFAFPRAGNLGNNIGEVNNNEGDLQPSSVVEPGEVG  211
            E+ R P +E+E P +     + A+  A     +LG   G  N +EG       +   ++G
Sbjct  44   EEPRAPWVEQEGPEYWEEQTRIAKANAQTDRVDLGTLRGYYNQSEGGSHTIQWMYGCDLG  103

Query  212  PAVWLGHAQNDDASDGLRFTVLNEDGN--EEPKLSRQSNQEDWK  253
            P   L H  + DA DG  +  LNED        ++ Q+ Q  W+
Sbjct  104  PDGRLLHGYHQDAYDGKDYIALNEDLRSWTAADIAAQNTQRRWE  147


>gb|AEG89315.1| MHC class I antigen [Macaca nemestrina]
Length=231

 Score = 38.1 bits (87),  Expect = 9.9, Method: Compositional matrix adjust.
 Identities = 24/82 (29%), Positives = 38/82 (46%), Gaps = 0/82 (0%)

Query  155  RTPLIEEEQPGFIHPSQQSAQQFAFPRAGNLGNNIGEVNNNEGDLQPSSVVEPGEVGPAV  214
            R P +E+E P +   + +SA+  A    GNL   +   N +EG      ++   ++GP  
Sbjct  72   RAPWMEQEGPEYWDRNTRSAKGHAQTHRGNLRTLLRYYNQSEGGSHTVQIMSGCDLGPDG  131

Query  215  WLGHAQNDDASDGLRFTVLNED  236
             L    +  A DG  +  LNED
Sbjct  132  RLLRGYHQHAYDGKDYIALNED  153


>dbj|BAK74794.1| MHC class I antigen, partial [Macaca fascicularis]
Length=213

 Score = 38.1 bits (87),  Expect = 9.9, Method: Compositional matrix adjust.
 Identities = 24/82 (29%), Positives = 38/82 (46%), Gaps = 0/82 (0%)

Query  155  RTPLIEEEQPGFIHPSQQSAQQFAFPRAGNLGNNIGEVNNNEGDLQPSSVVEPGEVGPAV  214
            R P +E+E P +   + +SA+  A    GNL   +   N +EG      ++   ++GP  
Sbjct  60   RAPWMEQEGPEYWDRNTRSAKGHAQTHRGNLRTLLRYYNQSEGGSHTVQIMSGCDLGPDG  119

Query  215  WLGHAQNDDASDGLRFTVLNED  236
             L    +  A DG  +  LNED
Sbjct  120  RLLRGYHQHAYDGKDYIALNED  141


Lambda      K        H        a         alpha
   0.309    0.130    0.395    0.792     4.96 

Gapped
Lambda      K        H        a         alpha    sigma
   0.267   0.0410    0.140     1.90     42.6     43.6 

Effective search space used: 3475736195593


  Database: nr
    Posted date:  Sep 23, 2015 12:05 AM
  Number of letters in database: 26,053,659,533
  Number of sequences in database:  71,551,133


Matrix: BLOSUM62
Gap Penalties: Existence: 11, Extension: 1
Neighboring words threshold: 11
Window for multiple hits: 40
```
